# Concurrent Kimura disease and lupus nephritis: A case report

**DOI:** 10.1097/MD.0000000000005086

**Published:** 2016-10-14

**Authors:** Haitao Wang, Fang Fang, Ying Sun, Songlan Wang, Yonghui Mao

**Affiliations:** a Department of Nephrolgy; b Department of Pathology, Beijing Hospital, National Center of Gerontology, China.

**Keywords:** case report, Kimura disease, lupus nephritis

## Abstract

**Background:**

Kimura disease is a rare chronic inflammatory disorder with peripheral eosinophilia and elevated serum IgE and is also frequently complicated by nephropathy.

**Methods:**

We report a rare case of Kimura disease concomitant with lupus nephritis in a 72-year old male patient with recurrent unexplained lymphadenopathy, renal lesions, and immunologic abnormalities.

**Results:**

The patient was successfully managed with gamma immunoglobulin, intravenous pulse methylprednisolone therapy, hydroxychloroquine, and prednisone.

**Conclusion:**

This is the first report of a case of Kimura disease concomitant with lupus nephritis and highlights the importance of considering lupus nephritis as a possible concurrent disease in patients with Kimura disease that have immunologic abnormalities.

## 1 Introduction

Kimura disease, or eosinophilic lymphoid granuloma, is a rare chronic inflammatory disorder that is characterized by angiolymphoid proliferation with peripheral eosinophilia and elevated serum IgE. It often produces subcutaneous nodules in the head and neck and frequently involves regional lymph nodes. Kimura disease is also frequently complicated by nephropathy, particularly membranous nephropathy.^[1–7]^ Systemic lupus erythematosus (SLE)^[8]^ is a multifactorial autoimmune disease that involves multiple organs in the body and is characterized by production of a variety of autoantibodies against self-antigens.^[9]^ Lupus nephritis affects 40% to 80% of SLE patients.^[10]^ Here, we report a rare case of Kimura disease concomitant with lupus nephritis and HBV-GN.

## 2 Case report

A 72-year old man was admitted to our hospital on October 15, 2014 because of generalized arthralgia and muscle pain for 1 month and worsening oral ulcer and body rashes for 3 weeks and fever for 5 days. The patient underwent left submandibular lymphadenectomy in 1965 because of enlarged lymph nodes after a mosquito bite. His eosinophil count was 4.7 × 10^9^/L (35%). He further received right submandibular lymphadenectomy in 1968 and supraclavicular lymphadenectomy in 1969 because of recurrent generalized lymph node enlargement. Pathological examination revealed eosinophilic granuloma. The patient received no treatment except Chinese herbal remedies, but his symptoms were gradually lessened. His eosinophil count fluctuated between 0.53 and 1.38 × 10^9^/L (6.9%–17.9%).

**Figure 1:**
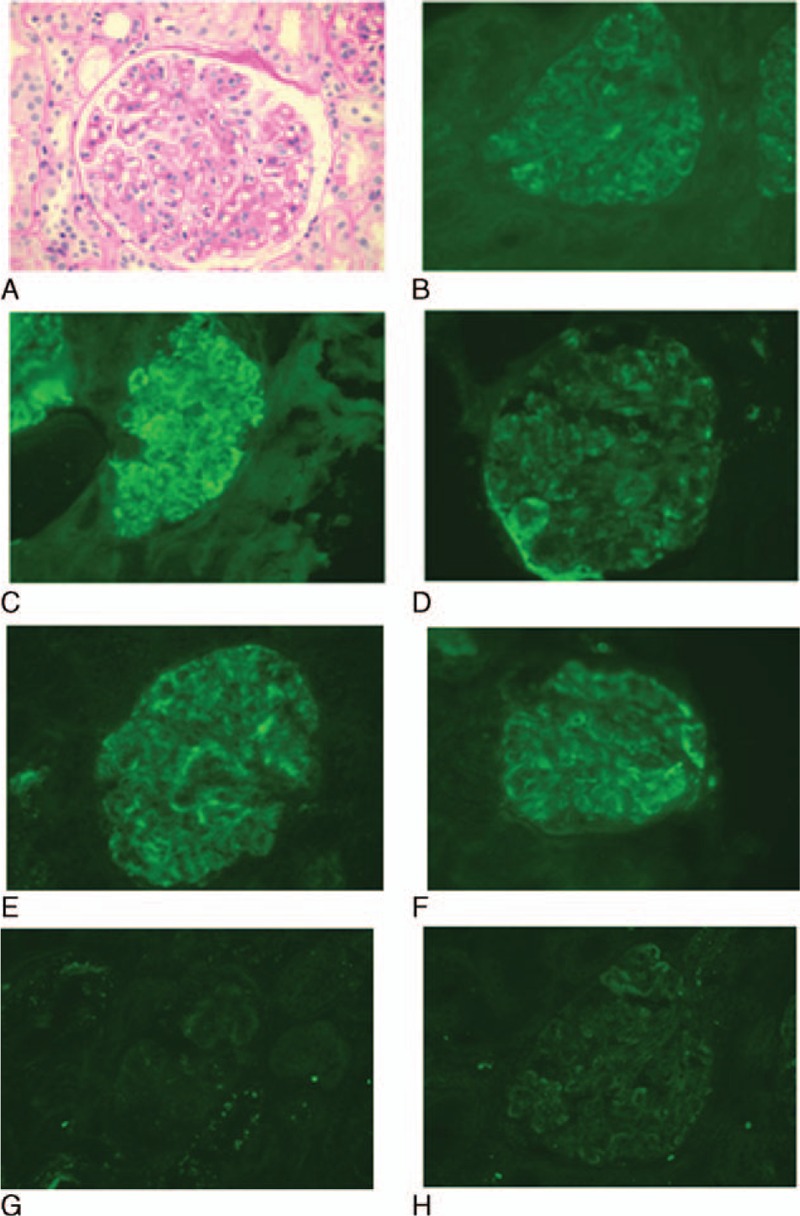
Initial renal biopsy in a 72-year old man with concurrent Kimura disease and lupus nephritis. (A) PAS staining reveals diffuse thickening of the basement membrane with appearance of tram tracks. The glomeruli show mild segmental proliferation of the mesangial cells and the matrix. The endothelial cells become swollen and proliferative. (B–G) Immunofluorescent microscopy reveals positive staining for IgA (B), IgG (C), IgM (D), C3 (E), and C1q (F) and false positive for HBsAg in kidney (G) and negative staining for HBcAg (H). Magnification, 400×. HBcAg = hepatitis B core antigen, HBsAg = hepatitis B surface antigen, Ig = immunoglobulin, PAS = periodic Acid–Schiff stain.

Routine urinalysis in March 2011 showed 20 to 30 deformed red blood cells per high power field and no proteinuria, and laboratory studies revealed a titer of 1:1280 for ANA and low C3 (29 mg/dL). The patient was otherwise asymptomatic and received no treatment. Two years later, proteinuria worsened with a 24-hour urinary protein of 1.7 g, and the patient was diagnosed with nephrotic syndrome and admitted. Physical examination revealed a blood pressure of 160/80 mm Hg. He had generalized lymph node enlargement. His eosinophil count was 1.13 × 10^9^/L and serum IgE was markedly elevated (149,000 IU/mL). The 24-hour urinary protein was 2.42 g and the patient was positive for ANA (1:320), anti-RNP antibody, and antiribosomal P antibody, but negative for antidouble stranded (ds) DNA antibody and anti-Sm antibody. He had a low C3 (48 mg/dL) and C4 (5 mg/dL). Serological study revealed HBsAg (−), HBeAg (−), HBsAg, and HBcAg(+) and HBV DNA was undetectable (<500 IU/mL). Renal biopsy revealed membranous nephropathy (Fig. 1A), immunofluorescence microscopy showed the presence of HbsAg (++) and HBcAg (++) and deposition of IgA (+++), IgG (++++), IgM (+++), C3 (++++), C4 (++), and C1q (+++) in the capillary loops and mesangium (Fig. 1B−H). The patient was diagnosed with HBV-GN and tentative Kimura disease. The patient refused lymph node biopsy. He was given cetirizine hydrochloride and olmesartan (20 mg/day). His proteinuria was lessened (0.08 g/24 hours) at a follow-up outpatient visit in April 2014.

**Figure 2:**
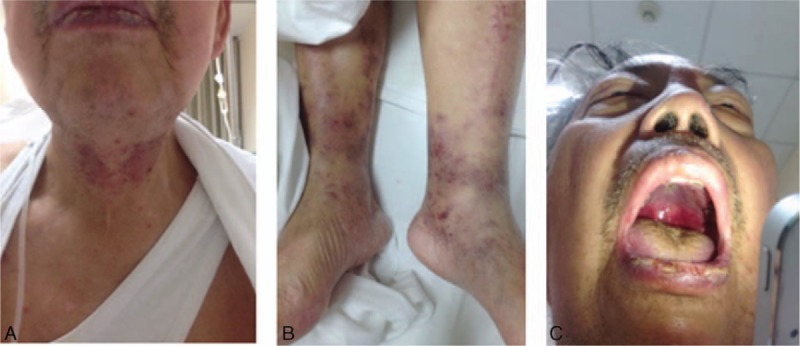
Red maculopapular eruptions are seen on the neck and forehead of the patient (A) and purpuras are also noticed in bilateral ankles (B). Ulcer and bleeding are present in the oral mucosa and tongue (C).

**Figure 3:**
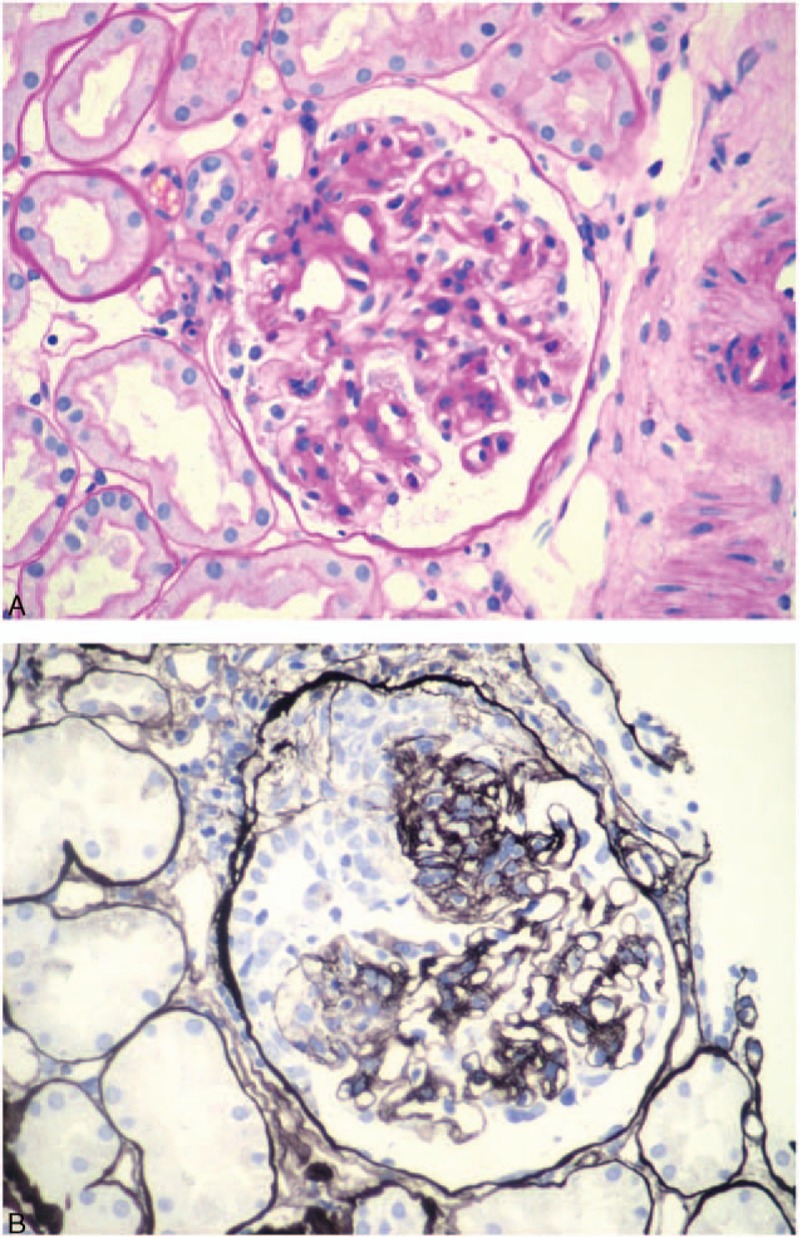
Repeat renal shows biopsy diffuse thickening of the basement membrane with appearance of tram tracks and the glomeruli exhibit mild to moderate segmental proliferation of the mesangial cells and the matrix (A, PAS staining). (B) PAM staining reveals diffuse thickening of the basement membrane and the presence of crescents. PAM = periodic acid-silver methenamine, PAS = periodic Acid–Schiff stain.

The patient developed generalized arthralgia and muscle pain 5 months later and was admitted to this hospital on October 15, 2014. At admission, the patient had a body temperature of 39 °C, and his blood pressure was 150/75 mm Hg. The patient was apparently in pain and had a puffy face. There were red maculopapular eruptions on the neck and forehead and purpura in bilateral ankles (Fig. 2A and B). Mild lip cyanosis was observed, a giant ulcer in the palate and bleeding in the oral mucosa and tongue were noted (Fig. 2C), and no crusted erosions existed on the lower lip. Moderate pitting edema was observed of both lower extremities and scattered purpuras were present at both ankles. Platelet count was 61 × 10^9^/L. The patient had a normal coagulation profile but bleeding time was not determined. Superficial lymph node was not palpated. Urinalysis revealed 40 to 45 leukocytes, 20 to 25 red blood cells, and 2 to 4 granular casts per high power field. The 24-hour urinary protein was 5.36 g. Chest CT revealed bilateral interstitial fibrosis. The patient received a final diagnosis of SLE according to the 2012 Systemic Lupus International Collaborating Clinic (SLICC SLE) criteria. The Systemic Lupus Erythematosus Disease Activity Index (SLEDAI) was 28. He was given gamma immunoglobulin (10 g/day for 5 days) and intravenous pulse methylprednisolone therapy (500 mg for 3 days), hydroxychloroquine (0.4 g/day), and prednisone 50 mg/day. He also received a single dose of intravenous cyclophosphamide (400 mg) when his lymphocytes fell below 1.0 × 10^9^/L. His temperature returned to normal 1 week after admission, oral ulcers improved, and maculopapular eruptions became lessened. Repeat renal biopsy showed 37 corpuscles under light microscope, 4 small cell crescents, and 1 small fiber crescent (Fig. 3). Mild to moderate segmental proliferation was observed of mesangial cells and matrix, and basement membrane became thickened and tram tracks were noted. Segmental necrosis of capillaries and dissolution of the mesangium were also seen. Immunofluorescence microscopy showed IgA (+++), IgG (++++), IgM (++), and C1q (+), which were mainly deposited in the capillary loop and mesangium, but no HbsAg and HBcAg were detected. The patient was diagnosed with class IV/V lupus nephritis. At the 10 week follow-up visit, his 24-hour urinary protein declined to 0.27 g, ANA tier was 1:320, and the patient was negative for anti-ds-DNA antibody. His SLEDAI was 2. His prednisone was tapered to 10 mg/day. Changes over time in leukocytes, eosinophils, immunoglobulins, autoantibodies, and complements in the patient is shown in Table 1, and changes over time in lymphocyte subsets are shown in Table 2.^[11]^

**Table 1:**
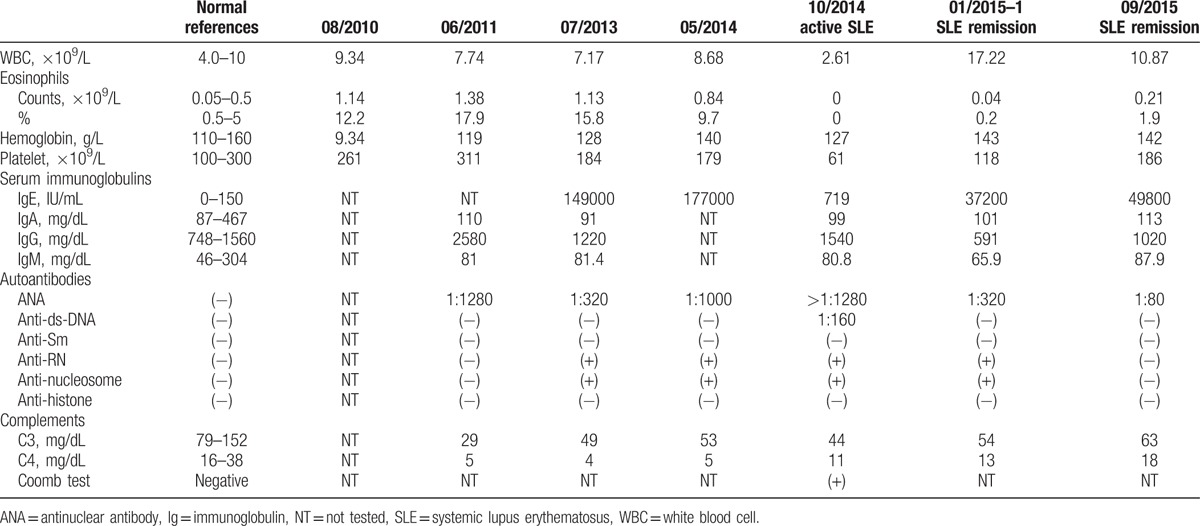
Changes over time in leukocytes, eosinophils, immunoglobulins, autoantibodies, and complements in the patient.

**Table 2:**
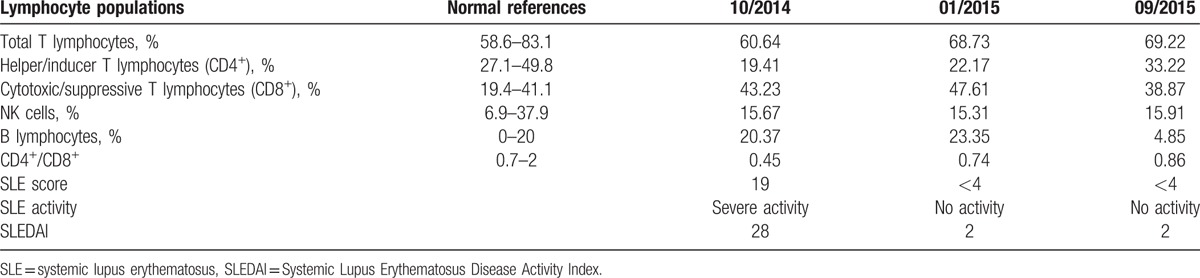
Changes over time in lymphocyte subsets.

This case report has been approved by Beijing Hospital ethics committee, and got the written consent of the patient.

## 3 Discussion

Kimura disease is mostly seen in young Asian adults and has a protracted course.^[12–14]^ As in this patient, almost all patients have marked IgE elevations and peripheral eosinophilia, and the percentage of eosinophils is as high as 10% to 20%.^[15,16]^ Diagnosis of the disease relies on pathologic study of lymph node or subcutaneous nodule biopsies demonstrating follicle-like growth and infiltration by eosinophils, which frequently form eosinophilic microabscess.^[17]^ The current case had recurrent episodes of lymphadenopathy over more than 40 years with peripheral eosinophilia and marked IgE elevations, and pathologic examination of excised lymph nodes suggested eosinophil infiltration and eosinophilic granuloma. The patient did not receive a diagnosis of Kimura disease then because of lack of understanding of Kimura disease. Though no biopsy was performed at the last admission, given the patient's history, he was diagnosed with Kimura disease.

The current case was not given a diagnosis of lupus nephritis at the 1st admission though his pathology suggested immune complex-mediated nephritis. As he had no evidence for the involvement of other organs and his renal tissues were tested positive for HBV, the patient was diagnosed with HBV-GN. Over the course of the illness, extrarenal manifestations became evident such as fever, skin eruptions, oral ulcers, and arthralgia. These manifestations, together with the immunologic abnormalities, led us to make a final diagnosis of SLE at the last admission.

Both Kimura disease and SLE may cause renal pathologies. Renal abnormalities are seen in 10% to 60% and proteinuria is reported in 15% to 18% of the cases of Kimura disease.^[2,18,19]^ Renal lesions, including membranous nephropathy, mesangial proliferative glomerulonephritis, and minimal change disease, typically appear months or even years after lymph node enlargement or development of subcutaneous nodules.^[3,20–22]^ In adults, membranous nephropathy is predominant and immunofluorescent microscopy mainly shows deposition of IgG and C3 and occasionally IgM and IgA, mostly in the capillary loop and less frequently in the mesangium. Infiltration into the interstitial by eosinophils provides an additional clue into the diagnosis of Kimura disease. Our patient was found to have membranous nephropathy on both admissions, but no eosinophil infiltration was observed. Immunofluorescent microscopy showed the presence of cellular and fiber crescents, which are not commonly seen in Kimura disease. On the other hand, these features are more in line with those of lupus nephritis (class V/IV). There has been no literature on concurrent Kimura disease and SLE though it has been reported that lupus nephritis patients have IgE elevations.^[23]^ In our case, IgE levels declined during active SLE, lymph nodes became shrunk and peripheral eosinophil counts decreased. When the patient had remission after immunosuppressive therapy, his IgE became elevated, suggesting that IgE elevations were likely due to Kimura disease, not SLE. The patient was very responsive to glucocorticoid therapy with more than 50% reduction in proteinuria at 4 weeks and achieved complete remission at week 10. This is different from class IV/V lupus nephritis.^[24]^

Currently, it is believed that Kimura disease is due toy type I hypersensitivity reaction or T cell-associated immune disorder.^[25]^ Some investigators showed that elevated CD4^+^ T cells and CD4^+^/CD8^+^ ratio in Kimura disease.^[26]^ Eosinophilia and IgE elevations suggest hyperfunctioning of Th2 cells. Ohta et al^[27]^ found no difference in the number of CD4^+^ and CD8^+^ between patients with Kimura disease and control patients, but the Th1/Th2 ratio was reduced while T_C_1/T_C_2 increased. Similarly, tissue damages as a result of abnormal production of lymphocytes, especially Th cells, and polyclonal activation of B cells play a critical role in the development of SLE. SLE patients also have abnormality in lymphocyte subset distribution, and recent evidence suggests that patients with active SLE have reduced CD4^+^ T cells and CD4^+^/CD8^+^ ratio, while the number of CD8^+^ cells remained unchanged or elevated.^[28,29]^ We determined T cell subsets of the patient during the active and remission stage of SLE and found that the number of CD4^+^ T cells and CD4^+^/CD8^+^ ratio were decreased while CD8^+^ cells increased, which is different from previous report on Kimura disease, but consistent with SLE. In addition, though CD4^+^ T cells and CD4^+^/CD8^+^ ratio did not recover to normal levels after SLE remission, but they were still higher than those during the active stage. We speculate that the abnormality of T cell subsets affected the Th1/Th2 ratio, leading to changes in serum IgE. T cell changes in patients with concurrent SLE and Kimura disease so far have remained undefined and should be further investigated.

The patient was diagnosed with HBV-GN on the 1st admission, but he did not meet the diagnostic criteria for SLE. The patient was tested positive for HBsAg and HBcAg in the renal tissues, which, however, were not detected in renal biopsy specimens at the last admission. Therefore, we did not include HBV-GN as a final diagnosis at the last admission. Previous studies have indicated that lupus nephritis tissues have a high HBV antigen detection rate,^[30]^ which could be as high as 50.6% in seronegative lupus nephritis patients,^[31]^ and the authors attributed this high rate to false positivity from indirect immunofluorescence. It remains likely that the current patient was tested falsely positive for HBV at the 1st admission and wrongly diagnosed with HBV-GN.

In conclusion, Kimura disease may coexist with SLE and immunologic abnormalities in the patient may be a consequence of both conditions.
